# Classification of *Fusarium*-Infected Korean Hulled Barley Using Near-Infrared Reflectance Spectroscopy and Partial Least Squares Discriminant Analysis

**DOI:** 10.3390/s17102258

**Published:** 2017-09-30

**Authors:** Jongguk Lim, Giyoung Kim, Changyeun Mo, Kyoungmin Oh, Hyeonchae Yoo, Hyeonheui Ham, Moon S. Kim

**Affiliations:** 1 Department of Agricultural Engineering, National Institute of Agricultural Sciences, Rural Development Administration, 310 Nongsaengmyeng-ro, Wansan-gu, Jeonju 54875, Korea; limjg@korea.kr (J.L.); giyoung@korea.kr (G.K.); yoonmine@korea.kr (K.O.); hyeonchae1@naver.com (H.Y.); 2 Microbial Safety Team, National Institute of Agricultural Sciences, Rural Development Administration, 166 Nongsaengmyeong-ro, Iseo-myeon, Wanju-gun 55365, Korea; hhham@korea.kr; 3 Environmental Microbial and Food Safety Laboratory, Agricultural Research Service, US Department of Agriculture, 10300 Baltimore Avenue, Beltsville, MD 20705, USA; Moon.Kim@ars.usda.gov

**Keywords:** *Fusarium*, near-infrared, spectroscopy, hulled barely, partial least squares-discriminant analysis

## Abstract

The purpose of this study is to use near-infrared reflectance (NIR) spectroscopy equipment to nondestructively and rapidly discriminate *Fusarium*-infected hulled barley. Both normal hulled barley and *Fusarium*-infected hulled barley were scanned by using a NIR spectrometer with a wavelength range of 1175 to 2170 nm. Multiple mathematical pretreatments were applied to the reflectance spectra obtained for *Fusarium* discrimination and the multivariate analysis method of partial least squares discriminant analysis (PLS-DA) was used for discriminant prediction. The PLS-DA prediction model developed by applying the second-order derivative pretreatment to the reflectance spectra obtained from the side of hulled barley without crease achieved 100% accuracy in discriminating the normal hulled barley and the *Fusarium*-infected hulled barley. These results demonstrated the feasibility of rapid discrimination of the *Fusarium*-infected hulled barley by combining multivariate analysis with the NIR spectroscopic technique, which is utilized as a nondestructive detection method.

## 1. Introduction

*Fusarium* (particularly *F*. *graminearum*) that occurs in the heads of cereal crops such as barley (*Hordeum vulgare L*.) and wheat (*Triticum aestivum L*.) has been reported to decrease the yield and degrade the quality, resulting in enormous economic losses to farmers [1]. The *Fusarium* pathogen grows rapidly between 10 and 25 °C in a high humidity environment due to heavy rainfall in the heading period of the cereal crops, leading to an increase in the occurrence frequency [2]. The pathogen wintering in seeds, straw, stubbles, and soil after harvest, becomes a primary inoculum, and molds formed from the primary symptoms scatter and rapidly spread [3,4,5]. Damaged ears develop a brown discoloration at the early stage, and the sheaths of ears become gradually covered with red conidiospores. In Korea, the incidence of *Fusarium* infection in barley increased greatly between 1963 and 1998. In 1998, the incidence of *Fusarium* infection damaged 39,202 hectares of fields, corresponding to 47.8% of the total cultivation area. Particularly, if mycotoxins such as deoxynivalenol (DON), nivalenol (NIV), and zearalenone (ZEA) produced by the *Fusarium* are mixed with the normal cereals and supplied in the finished product, this could cause intoxication of livestock as well as diseases such as vomiting, diarrhea, immunosuppression, and cancer in humans [6,7,8]. The most severe concern relating to *Fusarium* infection is that mycotoxins occurring due to *Fusarium* infection and remaining as a carcinogen in the feed could cause serious damage to livestock and humans [9]. Therefore, a preliminary inspection of barley, wheat, maize, and other cereal crops is essential to prevent *Fusarium* and mycotoxins from being introduced in the finished products.

Typical contamination inspections of *Fusarium* and mycotoxin use high performance liquid chromatography (HPLC), gas chromatography (GC), and enzyme-linked immunosorbent assay (ELISA). These destructive chemical analysis methods are demanding in terms of cost, time, and effort, and do not facilitate prompt detection due to small sample size and complicated pretreatment processes [10]. Nondestructive and rapid spectroscopic analysis techniques have emerged as alternatives to overcome these disadvantages.

Spectroscopic analysis techniques are nondestructive measurement methods that can be used for rapid quantitative analysis. These include NIR spectroscopy, Raman spectroscopy, and other spectroscopic methods according to light characteristics and analyte type, and can be used for pathogen detection as well as internal and external quality analysis of agricultural products [11]. NIR spectroscopic analysis uses the wavelength range from 700 nm to 2500 nm. Although the near-infrared region of the spectrum was discovered 160 years ago, its practical analytical applications were first performed by a U.S. chemist, Norris. In the 1960s, Norris proved the potential of NIR spectroscopy through quantitative analysis of moisture and protein content in wheat [12]. The later development of chemometric techniques expanded the applications of NIR spectroscopy. The chemometric technique analyzes wavelength-based data from a spectrometer to accurately predict the components in a short time [13]. Analysis methods such as principal component analysis (PCA), partial least squares (PLS), partial least squares-discriminant analysis (PLS-DA), and linear discriminant analysis (LDA) have been used for model development [14].

The NIR spectroscopic technique has recently been applied to detect mold and mycotoxin in cereal crops such as wheat, barley, and maize [15,16,17]. Delwiche used the NIR technique to discriminate between wheat *Fusarium* and other molds with accuracies of 95% and 98%, respectively [18]. Polder showed PLS results using a transmission image in the visible range to detect *Fusarium* head blight (FHB, also known as ‘scab’ or ear blight) [19]. Liu used a 1064-nm NIR laser to reduce the fluorescence that occurs in barley and wheat, in order to detect a low concentration of mycotoxin [20]. Gaspardo used the Fourier transform (FT)-NIR technique to predict the concentrations of the mycotoxins fumonisin (B_1_) and fumonisin (B_2_) in maize kernels [21].

This study aimed to develop a technique for discriminating between *Fusarium-*infected hulled barley and normal hulled barley by using a NIR spectrometer. For this purpose, various spectral pretreatments were applied to the reflectance spectra from 1175 nm to 2170 nm. The study was further aimed to nondestructively detect *Fusarium-*infected hulled barley by developing a PLS-DA model.

## 2. Materials and Methods

### 2.1. Materials

The samples used in this experiment were 515 kernels of hulled barley samples that were collected from five Korean provinces in 2014. The samples were divided into a control group and experimental groups, as shown in Table 1. The control group samples that were not infected with *Fusarium* in the preliminary investigation were 127 kernels of one group (JN151) collected from Jeonnam Province. The experimental group samples containing *Fusarium*-infected hulled barley include 80 kernels of one group (GN121) from Gyeongnam Province as well as 95 kernels, 108 kernels, and 105 kernels of three groups (JB021, JB061, and JB094) from Jeonbuk Province.

As shown in Table 2, the hulled barley samples used in the NIR spectroscopic experiments were stored as individual kernels in a storage box with a 10 × 10 grid structure, and refrigerated at 4 °C.

### 2.2. Near-Infrared Measurement System

**Figure 1:**
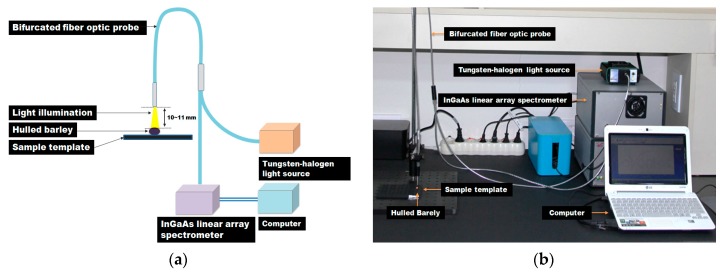
(**a**) Schematic diagram and (**b**) photo of the NIR measurement system for the discrimination of hulled barley infected with *Fusarium*.

The NIR measurement system, as shown in Figure 1, consists of an indium gallium arsenide (InGaAs) linear array spectrometer (AvaSpec-NIR256-2.2TC, Avantes BV Inc., Apeldoorn, The Netherlands) based on the symmetrical Czerny-Tunner optical design with a 50-mm focal length, a 10 W tungsten-halogen lamp light source (AvaLight-HAL, Avantes BV Inc.), a bifurcated fiber optic probe (FCR-7IR400-2-ME, Avantes BV Inc.) covering 350 nm to 2000 nm, a specimen stage, and a computer. As shown in Table 3, the NIR spectrometer is thermoelectrically cooled by a double-stage Peltier effect device, and has 256 light-sensitive pixels (50 × 500 μm pixel size, 3.4 nm average pixel interval) covering the spectral range from 1175 nm to 2170 nm. The light source provides illumination between 360 nm and 2500 nm, and uses a subminiature A (SMA) connector to maximize light coupling into light-conducting optic fibers with a diameter of up to 600 μm. The probe has a Y-style structure with a total length of 2 m, with 400-μm-diameter fibers.

Seven optic fibers are arranged on the stainless-steel tip (length 50 mm, diameter 6.35 mm) at the end of the probe. Among the seven-fiber optics, six optical fibers, which are arranged in a circle on the outer side, transmit the light from the light source to the hulled barley samples, and the optical fiber at the center transmits the light reflected from the hulled barley samples to the InGaAs spectrometer. The distance between the hulled barley samples and the probe end was maintained at 10 mm to 11 mm, vertically.

### 2.3. Medium Preparation for Fusarium Culture of Hulled Barley

After obtaining the NIR reflectance spectra, the hulled barley samples further underwent a culture experiment to visually determine whether a *Fusarium* infection was present. The medium used in this experiment was a mixture of materials from Difco^TM^ Potato Dextrose Agar (PDA, Difco Labs, Detroit, MI, USA) and Bacto^TM^ Agar (BD Biosciences, San Jose, CA, USA) to smoothly culture and easily solidify *Fusarium*. The medium was prepared by mixing 26 g/1000 mL of Difco^TM^ Potato Dextrose Agar and 5.6 g/1000 mL Bacto^TM^ Agar in distilled water, and then sterilized at high pressure. The solution was then cooled, and antibiotics that had been stored in a frozen state were injected into the medium solution to inhibit the growth of other molds. The antibiotics used in this experiment were neomycin sulfate (MP Biomedicals Inc., Solon, OH, USA) and streptomycin sulfate (Biosesang, Seongnam, Korea). Neomycin (0.5 g/50 mL) was dissolved into distilled water, further filtered through a 0.2 μm filter, and injected at a ratio of 12 mL per 1000 mL and 2.5 g/50 mL of streptomycin was dissolved in distilled water, further filtered through a 0.2 μm filter, and injected at a ratio of 20 mL per 1000 mL.

**Figure 2:**
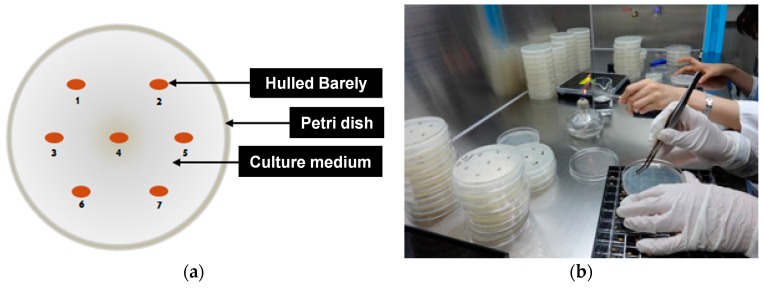
(**a**) Location and (**b**) configuration of kernels on Petri dish to culture *Fusarium* of hulled barley.

The medium solution was solidified in a Petri dish for 2 to 3 h. Subsequently, seven kernels of hulled barley samples were placed on each Petri dish, as shown in Figure 2. The Petri dishes were sealed with tape to prevent infection by foreign germs, and then incubated at 25 °C for 2 to 4 days in a darkened incubator. Any *Fusarium* growth was then classified visually.

### 2.4. Acquisition and Pretreatment of NIR Reflectance Spectra

**Figure 3:**
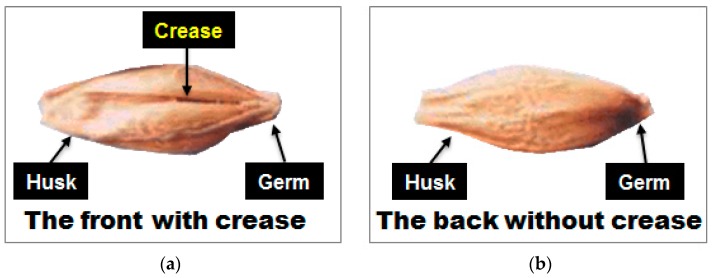
Denotation of (**a**) front side and (**b**) back side of hulled barley by the presence of the crease.

A hulled barley has an embryo in the lower part of the pericarp of the grain as well as a valley extending horizontally from the embryo, which is called the crease. In this experiment the side with the crease region was denoted as the front side and the opposite side as the back side, as shown in Figure 3. The measurement of one hulled barley kernel sample was repeated three times for the front side and for the back side, leading to a total of six spectra for each sample.

**Figure 4:**
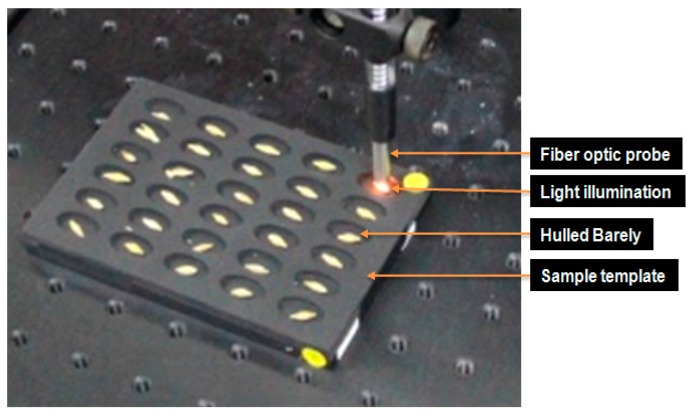
Probe and light illumination in NIR reflectance spectrum acquisition for hulled barley.

As shown in Figure 4, the hulled barley kernels were transferred to the optimal samples template for measurements. The integration time was 200 ms, and the spectra were cumulatively averaged 3 times. The smoothing pretreatment was applied to the basic reflectance spectra.

Reflectance spectral data obtained from hulled barley samples require calibration due to the irregular surface shape, and the data are pretreated using various mathematical methods to develop the best discriminant prediction model [22,23]. Several preprocessing techniques were applied to the obtained reflectance spectra to reduce the systematic noise and variation caused by the light source. The mathematical pretreatments used in this experiment include first-order derivative, second-order derivative, third-order derivative, mean normalization, maximum normalization, range normalization, mean scattering correction (MSC), baseline, and standard normal variate (SNV).

### 2.5. Fusarium Discrimination Prediction Model Development

In the PLS model, actually measured concentration information were used as dependent variables to develop a linear regression model with spectrum data, which are independent variables, and to predict actual concentration values [24,25,26,27]. PLS-DA is based on the classical partial least squares regression to construct prediction models [28]. In this regard, PLS-DA develops a regression model by designating the groups to be discriminated as dummy constants instead of concentration values as dependent variables. For a development of a PLS-DA model, this study designated as dependent variables with constant value ‘0’ the reflectance spectra obtained from hulled barley samples not infected with *Fusarium*, and as dependent variables with constant value ‘1’ the reflectance spectra obtained from hulled barley samples infected with *Fusarium* after the cultivation. A cross-validation method was applied to validate the PLS-DA models, and the accuracy of the developed models was determined by its coefficient of determination for calibration (R_C_^2^), the standard error of calibration (SEC), the coefficient of determination for validation (R_V_^2^), the standard error of prediction (SEP), and the optimal factor (F). Data pretreatment, model development, and validation using the obtained spectrum were performed by using multivariate data analysis software (Unscrambler v9.2, Camo Co., Oslo, Norway).

## 3. Results

### 3.1. Culture Results of Hulled Barley

#### 3.1.1. Culture Results of Hulled Barley Classified as Control Group

**Figure 5:**
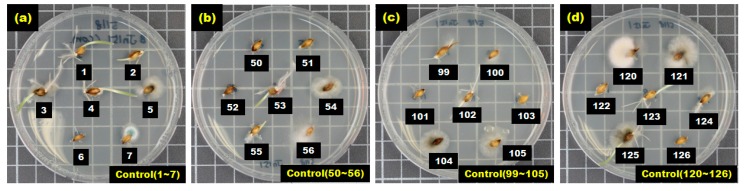
Culture results for hulled barley (JN151) in the control group, showing that *Fusarium* was not observed.

After seven hulled barley samples cultured in each Petri dish were incubated for between 2 and 4 days in an incubator, the occurrence of *Fusarium* was visually observed to determine whether a sample was infected. Figure 5 shows the selected culture results (1 to 7, 50 to 56, 99 to 105, and 120 to 126) for 127 hulled barley samples that were used in the control group, indicating that *Fusarium* spores were not observed.

#### 3.1.2. Culture Results of Hulled Barley Classified as Experimental Group

**Figure 6:**
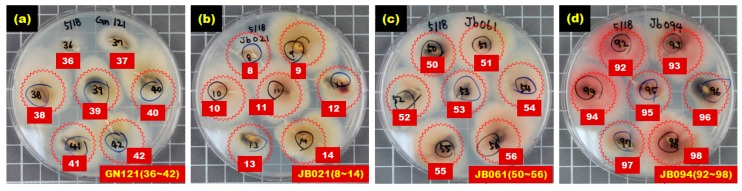
Culture results for hulled barley (GN121, JB021, JB061, JB094) in the experimental group in which *Fusarium* was observed.

Figure 6 shows the selected culture results for hulled barley samples in the *Fusarium*-infected sample groups (GN121, JB021, JB061, JB094), in which *Fusarium* spores were actively generated and dispersed within the Petri dish, indicating that these hulled barley samples were infected with *Fusarium*.

Table 4 summarizes the results of the culture tests on the control and experimental sample groups, and shows that some hulled barley in the infected sample groups did not develop *Fusarium*. In the GN121 sample group, 46 kernels were infected among the total 80 kernels. In the JB021 sample group, 82 kernels were infected among the total 95 kernels. Furthermore, in the JB061 and JB094 sample groups, 82 kernels of hulled barley samples among the 108 kernels, and 88 kernels among the 105 kernels were infected, respectively. To discriminate *Fusarium*-infected kernels by using the NIR spectroscopic technique, the kernels were divided into 127 kernels in the control group and 298 (46 + 82 + 82 + 88) *Fusarium*-infected kernels in the contaminated sample group.

### 3.2. Spectral Characteristics of Hulled Barley

**Figure 7:**
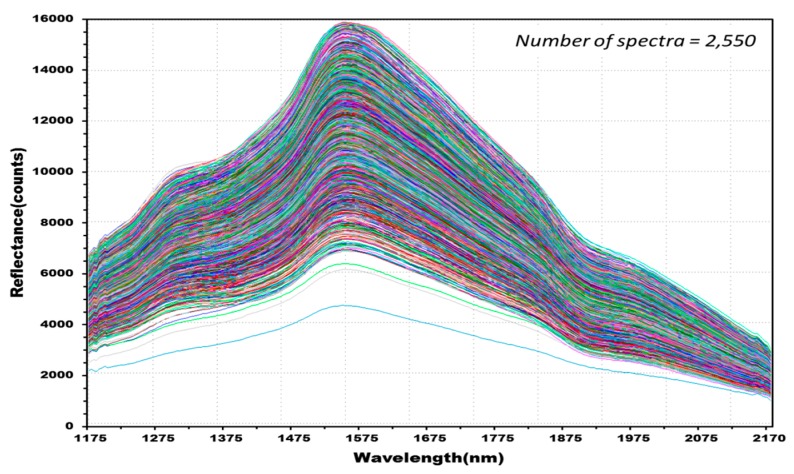
NIR reflectance spectra obtained from hulled barley samples (515 kernels) in both the control and experimental groups.

The experiment measured the NIR reflectance spectra of the total 515 kernels including 388 kernels in the four experimental groups classified as containing *Fusarium*-infected samples, and 127 kernels in the control group. A discriminant prediction model was developed using the NIR reflectance spectra. Figure 7 shows the 2550 reflectance spectra obtained by measuring both the front and back of each kernel three times. The maximum reflectance of the hulled barley was observed around 1575 nm, a rising peak was observed around 1315 nm, and the reflectance intensity then decreased around 1935 nm.

**Figure 8:**
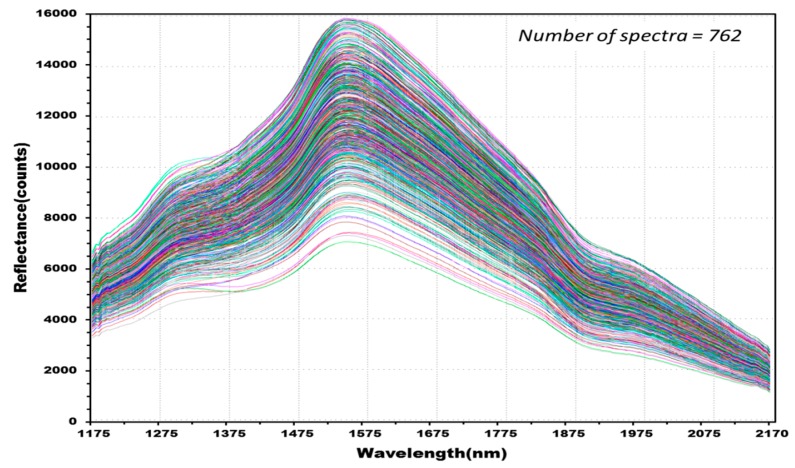
Total NIR reflectance spectra obtained from normal hulled barley samples (127 kernels) in the control group.

**Figure 9:**
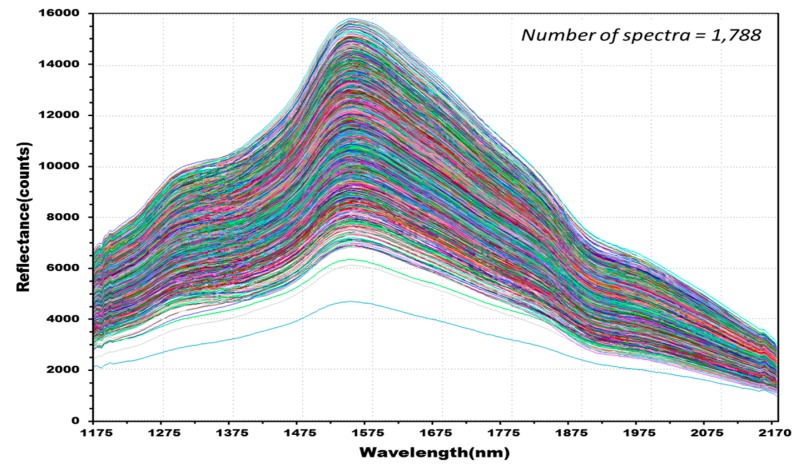
Total NIR reflectance spectra obtained from *Fusarium*-infected hulled barley samples (298 kernels) in the experimental group.

Figure 8 shows 762 reflectance spectra obtained from 127 kernels of normal hulled barley samples in the control group. Figure 9 shows 1788 reflectance spectra obtained from the 298 kernels infected with *Fusarium*. The raw reflectance spectra showed no difference in the reflectance spectrum intensity between the uninfected group and the group infected with *Fusarium*. Thus, it was difficult to discriminate the infection with the reflectance spectrum alone.

**Figure 10:**
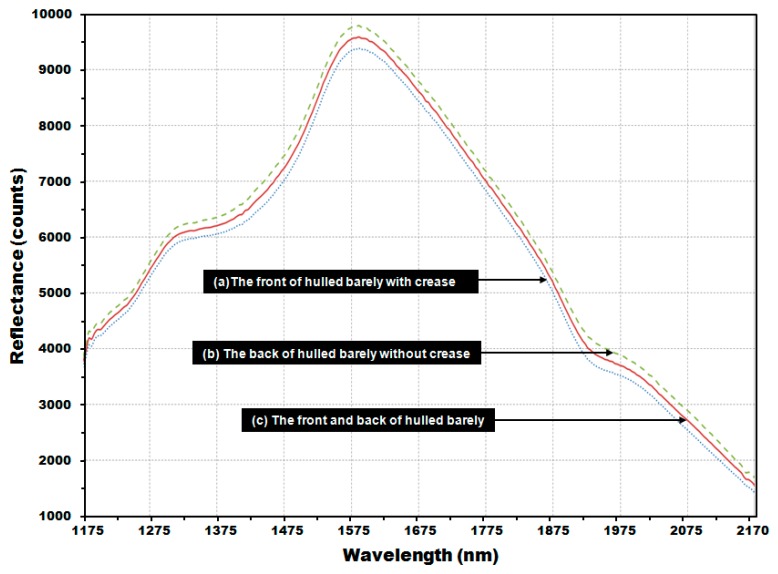
Average NIR reflectance spectra obtained from (**a**) the front side of the hulled barley with crease; (**b**) the back side of the hulled barley without crease; and (**c**) both front and back sides of the hulled barley.

Figure 10 shows the average reflectance spectra for 2550 reflectance spectra obtained from 510 kernels of hulled barley samples used to develop the PLS-DA model, comprised of (a) 1275 reflectance spectra obtained from the front side of the kernels (with crease), and (b) 1275 reflectance spectra obtained from the back-side samples (without crease). As shown in each average reflectance spectra, the reflectance intensity was overall higher on the back side (b) than on the front side (a). The reflectance difference could be due to a decrease in the quantity of reflectance because the valley formed by the crease scatters relatively more light. The average (c) of the total NIR reflectance spectra obtained from both the front side and the back side showed the median reflectance value between (a) and (b).

### 3.3. Prediction Results of PLS-DA Model for Fusarium-Infected Huske Barley

This study applied the PLS-DA method to discriminate *Fusarium*-infected hulled barley by using the reflectance spectra and the culture results of the hulled barley samples. Independent variables used for PLS-DA model development are the reflectance spectra obtained from hulled barley samples. As the dependent variables, the random dummy variables were designated as ‘0’ for the reflectance spectra obtained from the normal samples that were not infected with *Fusarium*, and the random dummy variables were designated as ‘1’ for the *Fusarium*-infected samples to develop a discriminant model.

Table 5 shows the results of the PLS-DA models developed by applying various pretreatments to the reflectance spectra of normal hulled barley samples from the control group as well as the reflectance spectra of *Fusarium*-infected hulled barley samples from the experimental group.

#### 3.3.1. Prediction Results of PLS-DA Model Using Reflectance Spectra Obtained from Front Side and Back Side of Hulled Barley

**Figure 11:**
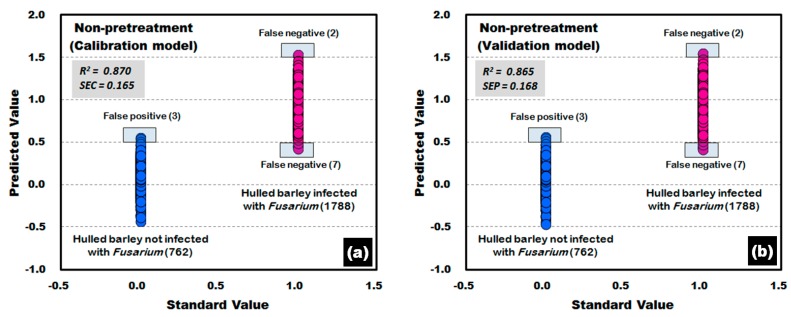
*Fusarium* discrimination calibration (**a**) and validation (**b**) results of the PLS-DA model developed using raw reflectance spectra obtained from the front side and back side of the hulled barley.

Figure 11 shows the calibration and validation results of a PLS-DA model developed without applying any pretreatment to the reflectance spectra obtained from the front side and back side of the hulled barley used in the experiment. In the validation model, three reflectance spectra among the 762 reflectance spectra obtained from the hulled barley not infected with *Fusarium*, were predicted (false positive) with a reference value of 0.5 or more, indicating an accuracy of 99.61%. On the other hand, two reflectance spectra among the 1788 reflectance spectra obtained from the hulled barley infected with *Fusarium*, were predicted (false negative) with a reference value of 1.5 or more, and 7 reflectance spectra were predicted (false negative) with a reference value of 0.5 or less, indicating an accuracy of 99.50%.

**Figure 12:**
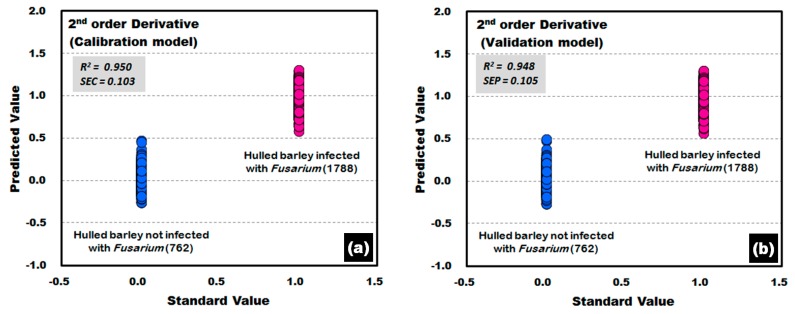
*Fusarium* discrimination calibration (**a**) and validation (**b**) results of PLS-DA model developed by applying the second-order derivative pretreatment to the reflectance spectra obtained from the front side and back side of hulled barley.

This study applied various spectrum pretreatments to improve the accuracy of the prediction model. Figure 12 shows the prediction results of the PLS-DA model, which was developed by applying the second-order derivative pretreatment to the raw reflectance spectra for the front and back sides of the hulled barley obtained from the control group and the experimental group. The results showed that the calibration and validation models had an excellent discrimination accuracy of 100%. In the validation model, to which the second-order derivative pretreatment was applied, R^2^ was improved to 0.948, which was better than that of the raw spectral data. Moreover, the SEP decreased to 0.105, all indicating that the spectrum pretreatment was effective.

#### 3.3.2. Prediction Results of PLS-DA Model Using Reflectance Spectra Obtained from Front Side of Hulled Barley

**Figure 13:**
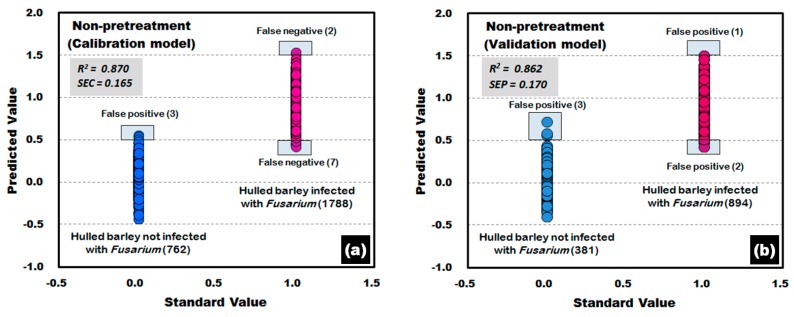
*Fusarium* discrimination calibration (**a**) and validation (**b**) results of PLS-DA model developed using the raw reflectance spectra obtained from the front side of hulled barley.

Figure 13 shows the results of PLS-DA model, which was developed by using the raw reflectance spectra that were obtained from the front side with crease on the hulled barley samples in both the control group and the experimental group. In the developed validation model, three reflectance spectra among the 381 reflectance spectra obtained from the front side of the hulled barley that were not infected with *Fusarium* were predicted with a reference value of 0.5 or more, indicating an accuracy of 99.21%. Among the 894 reflectance spectra obtained from the back side of the *Fusarium*-infected hulled barley, one reflectance spectrum was predicted as false negative with a reference value of 1.5 or more, and two reflectance spectra were predicted as false negative with a reference value of 0.5 or less, indicating an accuracy of 99.66%.

**Figure 14:**
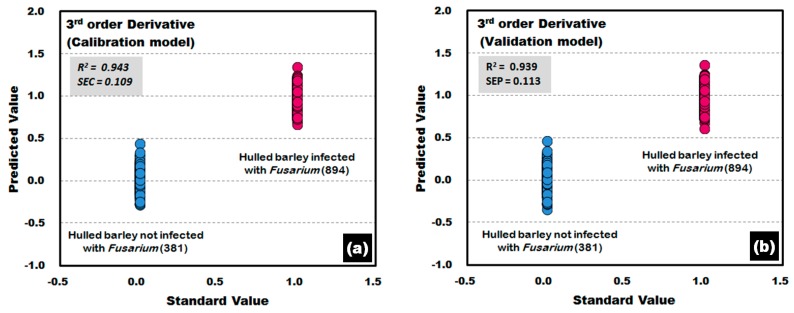
*Fusarium* discrimination calibration (**a**) and validation (**b**) results of PLS-DA model developed by applying the third-order derivative pretreatment to the reflectance spectra obtained from the front side of hulled barley.

Figure 14 shows the calibration and validation results of the PLS-DA model that was developed by applying the third-order derivative pretreatment to the raw reflectance spectra obtained from the front side of the hulled barley, indicating 100% discrimination accuracy. The results of the developed validation model show that R^2^ was improved to 0.939 and that SEP decreased to 0.113.

#### 3.3.3. Prediction Results of PLS-DA Model Using Reflectance Spectra Obtained from the Back Side of the Hulled Barley

**Figure 15:**
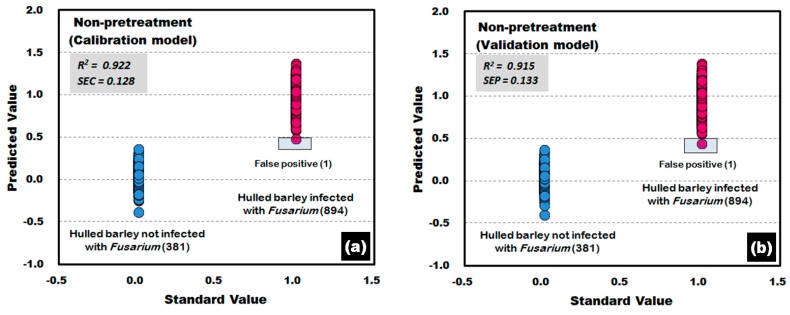
*Fusarium* discrimination calibration (**a**) and validation (**b**) results of PLS-DA model developed by using the raw reflectance spectra obtained from the back side of hulled barley.

Figure 15 shows the calibration and validation results of the PLS-DA model. To obtain the results, the raw reflectance spectra acquired from the back side of the hulled barley (without crease) was used to develop the PLS-DA model. The results showed an accuracy of 99.89% because one reflectance spectrum from each of the calibration and validation models deviated from the reference value (false positive) out of the 894 reflectance spectra for *Fusarium*-infected hulled barley samples.

**Figure 16:**
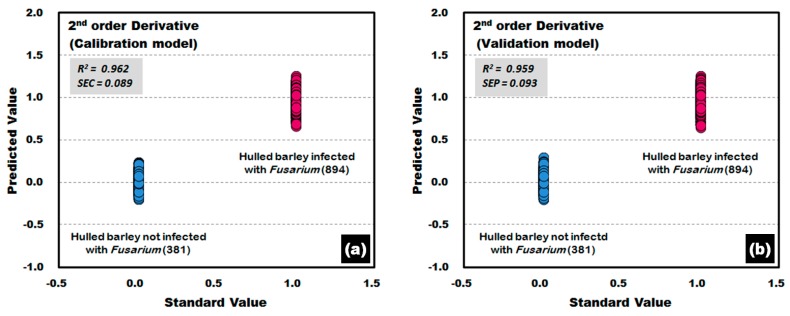
*Fusarium* discrimination calibration (**a**) and validation (**b**) results of the PLS-DA model developed by using the raw reflectance spectra obtained from the back side of the hulled barley.

The PLS-DA model was developed by applying the second-order derivative pretreatment acquired from the side of the hulled barley without the crease. The calibration and validation results of the PLS-DA model are shown in Figure 16. Of the 894 reflectance spectra for *Fusarium*-infected hulled barley samples, one reflectance spectrum from each of the calibration and validation models deviated from the reference value as a false positive. The results show an accuracy of 99.89%.

## 4. Conclusions and Outlook

This paper indicates potential advantages of NIR spectroscopy for the discrimination of *Fusarium*-infected Korean hulled barley. This study nondestructively discriminated *Fusarium*-infected hulled barley and normal hulled barley by using NIR spectroscopy from 1175 nm to 2170 nm to measure the reflectance spectrum intensity of the hulled barley. The study further applied various spectrum pretreatments to develop the discriminant prediction models and validate the performance and discrimination accuracy. 127 kernels of hulled barley samples not infected with *Fusarium* and 298 kernels confirmed with *Fusarium* spores by a culture test were used as experiment samples. Each measurement was repeated three times for both sides of the hulled barley kernels to obtain 2550 reflectance spectra. Various mathematical pretreatments were applied to the obtained reflectance spectra to minimize the instability of the light source as well as several changes that occur in the size and shape of the samples, and the discrimination performance and accuracy for each pretreatment were examined by using the PLS-DA method to develop discriminant prediction models. To verify the accuracy of the developed PLS-DA model, additional specimens should be obtained to verify the sensitivity and specificity. These studies using NIR reflectance spectroscopy indicate the potential of this spectroscopic technique for agricultural application in the near future.

In the *Fusarium* discrimination performance of the developed PLS-DA validation model by using the 2550 raw reflectance spectra, the discrimination accuracy for the normal hulled barley samples was 99.61% and the accuracy for *Fusarium*-infected hulled barley samples was 99.50%. Validation was performed to develop a technique for nondestructive prediction. After the application of secondary derivative pretreatment to improve discrimination accuracy, the results showed 100% discrimination accuracy for discriminating normal and *Fusarium*-infected hulled barley samples.

The PLS-DA validation prediction model developed by applying 1275 raw reflectance spectra obtained from the front side of the hulled barley, showed accuracies of 99.21% and 99.66% for discriminating the normal and *Fusarium*-infected hulled barleys, respectively. The PLS-DA model developed by applying the third-order derivative pretreatment showed 100% accuracy for classification. The discrimination results of the PLS-DA validation model developed by using the raw reflectance spectra obtained from the back side of the hulled barley (without crease) with the best discrimination prediction results, showed an accuracy of 100% for the normal samples and 99.89% for the *Fusarium*-infected samples. Furthermore, the results with the application of the second and the third-order derivative pretreatments showed 100% classification accuracy.

As shown in the results from this study, the PLS-DA prediction model, developed by applying the reflectance spectra obtained from the NIR spectroscopic equipment with the optimal mathematical pretreatment, is anticipated to achieve nondestructive and rapid detection of *Fusarium*-infected hulled barley.

## Figures and Tables

**Table 1:** Collection province and quantity of hulled barley samples used in experiment.

Province	Groups	Sample Group Based on Region	Number of Kernels
Jeonnam	Control group	JN151	127
Gyeongnam	Experimental group	GN121	80
Jeonbuk		JB021	95
		JB061	108
		JB094	105
Total		5	515

**Table 2:** Hulled barley samples separated by kernel into grid boxes according to groups.

Control Group		Experimental Groups			
JN151 (127)		GN121 (80)	JB021 (95)	JB061 (108)	JB094 (105)
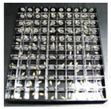	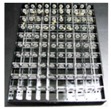	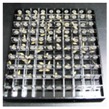	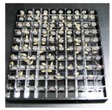	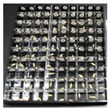	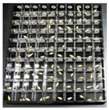

**Table 3:** Specifications of NIR spectrometer used for classification of *Fusarium*-infected hulled barley.

Model (Manufacturer)	Ava Spec-NIR256-2.2TEC (Avantes BV, Apeldoorn, The Netherlands)
Appearance	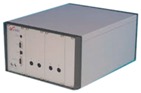
Spectral range	1175–2170 nm
Detection sensor	InGaAs linear array
Pixel pitch	3.4 nm
Pixel size	50 × 500 μm
Total pixel count	256
Minimum exposure time	1 ms
Signal to noise ratio	4100:1
PC interface	USB 2.0
Dimensions	315 × 235 × 135 mm
Weight	5.1 kg

**Table 4:** Culture results for control group and experimental group of hulled barley samples infected with *Fusarium*.

Experimental Group	Number of Grains (Sample Group)	Culture Results	
		^a^ NIF	^b^ IF
Hulled barley group classified as not infected with *Fusarium*	127 (JN151)	**127**	0
Hulled barley group classified as infected with *Fusarium*	80 (GN121)	34	**46**
	95 (JB021)	13	**82**
	108 (JB061)	26	**82**
	105 (JB094)	17	**88**

Note: ^a^ NIF, hulled barley not infected with *Fusarium*; ^b^ IF, hulled barley infected with *Fusarium*.

**Table 5:** Performance of the PLS-DA calibration and validation models for *Fusarium*-infected hulled barley samples as well as classification accuracy for normal and infected hulled barleys.

Pretreatment	^a^ F	^b^ PC		^c^ PV			
		R_C_^2^	SEC	R_V_^2^	SEP	^d^ CCR	
						NIF	IF
***The front and back measurement results of hulled barley***							
Non-pretreatment	17	0.870	0.165	0.865	0.168	99.61	99.50
1st order Derivative	14	0.924	0.126	0.919	0.131	99.87	**100.00**
2nd order Derivative	**10**	**0.950**	**0.103**	**0.948**	**0.105**	**100.00**	**100.00**
3rd order Derivative	**9**	**0.942**	**0.110**	**0.941**	**0.112**	**100.00**	**100.00**
Mean Normalization	17	0.868	0.166	0.863	0.170	99.87	99.55
Maximum Normalization	17	0.865	0.168	0.860	0.172	99.74	99.38
Range Normalization	17	0.860	0.171	0.855	0.175	99.87	99.44
MSC	16	0.852	0.176	0.846	0.180	99.87	99.22
Baseline	14	0.838	0.184	0.835	0.186	99.48	98.99
SNV	17	0.853	0.176	0.847	0.179	99.87	99.16
***The front measurement results with crease of hulled barley***							
Non-pretreatment	17	0.872	0.164	0.862	0.170	99.21	99.66
1st order Derivative	15	0.938	0.114	0.929	0.122	99.74	**100.00**
2nd order Derivative	**10**	**0.948**	**0.104**	**0.944**	**0.108**	**99.74**	**100.00**
3rd order Derivative	**9**	**0.943**	**0.109**	**0.939**	**0.113**	**100.00**	**100.00**
Mean Normalization	16	0.858	0.172	0.847	0.179	99.48	99.78
Maximum Normalization	16	0.854	0.175	0.843	0.182	99.48	99.66
Range Normalization	17	0.858	0.173	0.847	0.179	99.48	99.33
MSC	16	0.845	0.180	0.833	0.187	99.21	99.22
Baseline	17	0.863	0.169	0.853	0.176	99.48	99.66
SNV	17	0.846	0.180	0.834	0.187	99.21	99.22
***The back measurement results without crease of hulled barley***							
Non-pretreatment	17	0.922	0.128	0.915	0.133	**100.00**	99.89
1st order Derivative	12	0.941	0.111	0.933	0.119	**100.00**	99.89
2nd order Derivative	**10**	**0.962**	**0.089**	**0.959**	**0.093**	**100.00**	**100.00**
3rd order Derivative	**9**	**0.954**	**0.098**	**0.951**	**0.102**	**100.00**	**100.00**
Mean Normalization	13	0.881	0.158	0.874	0.163	**100.00**	99.66
Maximum Normalization	13	0.878	0.160	0.871	0.165	**100.00**	99.55
Range Normalization	13	0.877	0.160	0.871	0.165	99.74	99.55
MSC	14	0.887	0.154	0.879	0.159	**100.00**	99.66
Baseline	14	0.897	0.147	0.890	0.152	**100.00**	99.78
SNV	14	0.878	0.160	0.871	0.165	**100.00**	99.55

Notes: ^a^ F, number of factors; ^b^ PC, performance of calibration; ^c^ PV, performance of validation; ^d^ CCR, correct classification rate.
